# Supplementary material for: Cross-asset momentum and the hybrid fund transmission mechanism in China’s stock and bond markets
Source: PLoS One. 2024 Mar 21;19(3):e0300781. doi: 10.1371/journal.pone.0300781 (PMC10956777; doi:10.1371/journal.pone.0300781)
Supplement: S1 Appendix — (DOCX) [file pone.0300781.s001.docx]

We have introduced a moderation effect model to explore the moderating role of hybrid fund indices in the cross-asset momentum transmission process. This serves as an additional analysis to complement our study.

We formulated the following model to assess the moderating effect of fund indices in the transmission of bond market momentum to the stock market:

$${{STOCK}_{t,t+4}=\gamma_{0}+\gamma_{1}FUND}_{t-4,t-1}+\gamma_{2}{CUMBOND}_{t-52,t-1}+\gamma_{3}{FUND}_{t-4,t-1}\times{CUMBOND}_{t-52,t-1}$$

$$+\gamma_{4}{CUMSTOCK}_{t-52,t-1}+\gamma_{5}{FUND}_{t-4,t-1}\times{CUMSTOCK}_{t-52,t-1}$$

$+\gamma_{6}{SHIBOR}_{t,t+4}+{\gamma_{7}M2}_{t,t+4}+\gamma_{8}{CPI}_{t,t+4}+\gamma_{9}{PMI}_{t,t+4}+\varepsilon_{t}$ (A1)

In Equation A1, the dependent variable ${STOCK}_{t,t+4}$ represents the future one-month return of the stock market index. The ${FUND}_{t-4,t-1}$ represents the past one-month return of the fund index and the ${CUMBOND}_{t-52,t-1}$ signifies the cumulative return of the bond market over the past 52 weeks, reflecting momentum in bond market returns.

The term ${FUND}_{t-4,t-1}\times{CUMBOND}_{t-52,t-1}$ represents the interaction term between the fund index and the cumulative return of the bond market. This term indicates the moderating effect of hybrid fund indices on bond market momentum in the transmission to the stock market.

The control variable ${CUMSTOCK}_{t-52,t-1}$ is included for stock market momentum, while ${FUND}_{t-4,t-1}\times{CUMSTOCK}_{t-52,t-1}$ serves as the control variable for the fund index and stock market momentum interaction. Additional control variables encompass macro factors that could potentially influence the regression results.

The interpretation of Equation A1 unfolds as follows: Given the significant $\gamma_{2}$ for ${CUMBOND}_{t-52,t-1}$ (indicating a significant impact of bond market momentum on the stock market), our primary focus shifts to $\gamma_{3}$. A statistically significant $\gamma_{3}$ would signify that the fund indices play a moderating role in transmitting bond market momentum to the stock market.

To delve into specifics, when $\gamma_{2}$ is significantly positive, a concurrently significant positive $\gamma_{3}$ suggests that the fund indices amplify the positive impact of bond market momentum on the stock market. Conversely, a significantly negative $\gamma_{3}$ would indicate that hybrid fund indices impede the positive influence of bond market momentum on the stock market.

Concerns regarding the potential correlation between the moderating variable ${FUND}_{t-4,t-1}$ and the primary variable ${CUMBOND}_{t-52,t-1}$ may arise. The coefficient $\gamma_{3}$ of the interaction term ${FUND}_{t-4,t-1}\times{CUMSTOCK}_{t-52,t-1}$ could potentially reflect the indirect impact of the primary variable ${CUMBOND}_{t-52,t-1}$ on the dependent variable ${STOCK}_{t,t+4}$ (i.e., the influence of the square term of ${CUMBOND}_{t-52,t-1}$) rather than the moderation effect of ${FUND}_{t-4,t-1}$ itself.

We incorporate ${FUND}_{t-4,t-1}$ in Equation A1 to address this concern. The coefficient $\gamma_{1}$ of ${FUND}_{t-4,t-1}$ assists in interpreting the meaning of $\gamma_{3}$. If $\gamma_{1}$ is found to be insignificant, it implies that the primary variable ${CUMBOND}_{t-52,t-1}$ cannot indirectly influence the dependent variable ${STOCK}_{t,t+4}$ through its impact on ${FUND}_{t-4,t-1}$. In this scenario, we conclude that $\gamma_{3}$ reflects the moderation effect of hybrid fund indices on bond market momentum.

Conversely, if $\gamma_{1}$ is significant, we cannot dismiss the possibility that ${CUMBOND}_{t-52,t-1}$ indirectly affects ${STOCK}_{t,t+4}$ through its influence on ${FUND}_{t-4,t-1}$, and we cannot confirm the moderation effect of hybrid funds.

Similarly, we formulated the following model to assess the moderating effect of fund indices in the transmission of stock market momentum to the bond market:

$${{BOND}_{t,t+4}=\gamma_{0}+\gamma_{1}FUND}_{t-4,t-1}+\gamma_{2}{CUMSTOCK}_{t-52,t-1}+\gamma_{3}{FUND}_{t-4,t-1}\times{CUMSTOCK}_{t-52,t-1}$$

$$+\gamma_{4}{CUMBOND}_{t-52,t-1}+\gamma_{5}{FUND}_{t-4,t-1}\times{CUMBOND}_{t-52,t-1}$$

$+\gamma_{6}{SHIBOR}_{t,t+4}+{\gamma_{7}M2}_{t,t+4}+\gamma_{8}{CPI}_{t,t+4}+\gamma_{9}{PMI}_{t,t+4}+\varepsilon_{t}$ (A2)

Table A1 presents the outcomes of Equation A1.

Firstly, for all four types of hybrid funds, the insignificance of $\gamma_{1}$ suggests that the past one-month return of the fund index does not forecast the future one-month return of the stock market index. This dismisses the possibility of the primary variable ${CUMBOND}_{t-52,t-1}$ indirectly influencing the dependent variable ${STOCK}_{t,t+4}$ by impacting ${FUND}_{t-4,t-1}$ (as indicated in the main results Table 9, where ${FUND}_{t,t+4}$ and ${STOCK}_{t,t+4}$ show significant correlation as they pertain to the same period). Furthermore, the coefficients for the control variables ${CUMSTOCK}_{t-52,t-1}$ and ${FUND}_{t-4,t-1}\times{CUMSTOCK}_{t-52,t-1}$ are also deemed insignificant.

Secondly, the significantly positive $\gamma_{2}$ indicates a favorable impact of bond market momentum on the stock market.

Lastly, the significantly positive $\gamma_{3}$ across all four types of hybrid funds signifies that in the process of bond market momentum transmission to the stock market, these hybrid fund indices amplify the positive impact of bond market momentum on the stock market.

Notably, the Wind Partial Bond Fund Index exhibits the most substantial moderating effect on bond market momentum, with a coefficient of 0.054. Intuitively, funds of this type maintain the highest bond allocation. Funds with higher bond allocations experience more excellent benefits during consistent bond price increases. Consequently, these funds rebalance their stock-bond asset ratios, allocating more capital to the stock market.

Table A2 reports the results of Equation A2.

Firstly, across all four types of hybrid funds, the insignificance of $\gamma_{1}$ suggests that the past one-month return of the fund index does not forecast the future one-month return of the bond market index. This dismisses the possibility of the primary variable ${CUMSTOCK}_{t-52,t-1}$ indirectly influencing the dependent variable ${BOND}_{t,t+4}$ by impacting ${FUND}_{t-4,t-1}$. The coefficient for the control variable ${CUMBOND}_{t-52,t-1}$ is positive at the 10% significance level, but the coefficient for the interaction term ${FUND}_{t-4,t-1}\times{CUMBOND}_{t-52,t-1}$ is deemed insignificant.

Secondly, the significantly negative $\gamma_{2}$ suggests a negative impact of stock market momentum on the bond market.

Lastly, $\gamma_{3}$ is significantly negative for all four types of hybrid funds, indicating that in the process of stock market momentum transmission to the bond market, hybrid fund indices amplify the negative impact of stock market momentum on the bond market.

In contrast to the findings reported in Table A1, it is evident that the Wind Partial Bond Fund Index demonstrates the least impactful moderating effect on stock market momentum at this juncture. This is indicated by the smallest absolute coefficient value, primarily attributed to these funds maintaining the lowest allocation to stocks. In periods of consistent stock price increases, these funds encounter restricted gains. Furthermore, due to the regulatory constraints associated with portfolio allocation ratios for these funds, their ability to enhance stock holdings by decreasing bond holdings is limited.

**Table A1. Result of the hybrid fund moderation on cross-asset momentum: from the bond to the stock market.**

| **Dependent variable:** $\boldsymbol{STOCK}_{\boldsymbol{t}\boldsymbol{,t+4}}$ | | | | |
| --- | --- | --- | --- | --- |
|  | **(1)** | **(2)** | **(3)** | **(4)** |
| $\boldsymbol{FUND}_{\boldsymbol{t}\boldsymbol{-4,t-1}}$ | **CSI Fund**  **Fund Index** | **Wind Partial**  **Stock**  **Fund Index** | **Wind Flexible**  **Fund Index** | **Wind Partial Bond**  **Fund Index** |
|  | 0.005 | 0.004 | -0.009 | 0.050 |
|  | 0.031 | 0.023 | 0.034 | 0.101 |
| $\boldsymbol{CUMBOND}_{\boldsymbol{t}\boldsymbol{-52,t-1}}$ | 0.154*** | 0.154*** | 0.157*** | 0.143*** |
|  | 0.039 | 0.046 | 0.047 | 0.046 |
| $\boldsymbol{FUND}_{\boldsymbol{t}\boldsymbol{-4,t-1}}\boldsymbol{\times}$  $\boldsymbol{CUMBOND}_{\boldsymbol{t}\boldsymbol{-52,t-1}}$ | 0.027** | 0.031** | 0.032** | 0.054* |
|  | 0.012 | 0.014 | 0.015 | 0.030 |
| $\boldsymbol{CUMSTOCK}_{\boldsymbol{t}\boldsymbol{-52,t-1}}$ | 0.002 | 0.002 | 0.002 | 0.002 |
|  | 0.004 | 0.004 | 0.004 | 0.004 |
| $\boldsymbol{FUND}_{\boldsymbol{t}\boldsymbol{-4,t-1}}\boldsymbol{\times}$  $\boldsymbol{CUMSTOCK}_{\boldsymbol{t}\boldsymbol{-52,t-1}}$ | 0.000 | 0.000 | 0.002 | -0.001 |
|  | 0.000 | 0.000 | 0.005 | 0.002 |
| $\boldsymbol{SHIBOR}_{\boldsymbol{t}\boldsymbol{,t+4}}$ | 0.001 | 0.002 | -0.002 | 0.011 |
|  | 0.113 | 0.112 | 0.112 | 0.113 |
| ${\boldsymbol{M}\boldsymbol{2}}_{\boldsymbol{t}\boldsymbol{,t+4}}$ | -0.587 | -0.593 | -0.535 | -0.703 |
|  | 0.547 | 0.547 | 0.547 | 0.547 |
| $\boldsymbol{CPI}_{\boldsymbol{t}\boldsymbol{,t+4}}$ | 0.213 | 0.148 | 0.160 | 0.147 |
|  | 0.284 | 0.284 | 0.284 | 0.285 |
| $\boldsymbol{PMI}_{\boldsymbol{t}\boldsymbol{,t+4}}$ | -0.001 | 0.010 | -0.008 | -0.014 |
|  | 0.140 | 0.139 | 0.140 | 0.139 |
| **Intercept** | -0.450 | -0.448 | -0.451 | -0.444 |
|  | 0.481 | 0.481 | 0.481 | 0.480 |
| **Adjusted R^2^** | 0.014 | 0.011 | 0.011 | 0.013 |
| **Observations** | 760 | 760 | 760 | 760 |

This table reports the result of the dependent variable ${STOCK}_{t,t+4}$.

Robust *t*-statistics are displayed in parentheses. *** indicates the 1% significance level; ** indicates the 5% significance level; * indicates the 10% significance level.

**Table A2 Result of the hybrid fund moderation on cross-asset momentum: from the stock to the bond market.**

| **Dependent variable:** $\boldsymbol{BOND}_{\boldsymbol{t}\boldsymbol{,t+4}}$ | | | | |
| --- | --- | --- | --- | --- |
|  | **(1)** | **(2)** | **(3)** | **(4)** |
| $\boldsymbol{FUND}_{\boldsymbol{t}\boldsymbol{-4,t-1}}$ | **CSI Fund**  **Fund Index** | **Wind Partial**  **Stock**  **Fund Index** | **Wind Flexible**  **Fund Index** | **Wind Partial Bond**  **Fund Index** |
|  | -0.001 | -0.001 | -0.002 | -0.004 |
|  | 0.002 | 0.002 | 0.002 | 0.007 |
| $\boldsymbol{CUMSTOCK}_{\boldsymbol{t}\boldsymbol{-52,t-1}}$ | -0.001*** | -0.003*** | -0.003*** | -0.002*** |
|  | 0.000 | 0.001 | 0.001 | 0.000 |
| $\boldsymbol{FUND}_{\boldsymbol{t}\boldsymbol{-4,t-1}}\boldsymbol{\times}$  $\boldsymbol{CUMSTOCK}_{\boldsymbol{t}\boldsymbol{-52,t-1}}$ | -0.009** | -0.008*** | -0.008** | -0.002** |
|  | 0.003 | 0.002 | 0.003 | 0.001 |
| $\boldsymbol{CUMBOND}_{\boldsymbol{t}\boldsymbol{-52,t-1}}$ | -0.005* | -0.004* | -0.005* | 0.006* |
|  | 0.005 | 0.003 | 0.004 | 0.003 |
| $\boldsymbol{FUND}_{\boldsymbol{t}\boldsymbol{-4,t-1}}\boldsymbol{\times}$  $\boldsymbol{CUMBOND}_{\boldsymbol{t}\boldsymbol{-52,t-1}}$ | -0.001 | -0.002 | -0.001 | -0.001 |
|  | 0.001 | 0.002 | 0.001 | 0.001 |
| $\boldsymbol{SHIBOR}_{\boldsymbol{t}\boldsymbol{,t+4}}$ | -0.025*** | -0.024*** | -0.028*** | -0.030*** |
|  | 0.007 | 0.007 | 0.008 | 0.008 |
| ${\boldsymbol{M}\boldsymbol{2}}_{\boldsymbol{t}\boldsymbol{,t+4}}$ | -0.006 | -0.005 | -0.005 | -0.003 |
|  | 0.039 | 0.039 | 0.040 | 0.040 |
| $\boldsymbol{CPI}_{\boldsymbol{t}\boldsymbol{,t+4}}$ | 0.022 | 0.022 | 0.023 | 0.019 |
|  | 0.021 | 0.021 | 0.021 | 0.021 |
| $\boldsymbol{PMI}_{\boldsymbol{t}\boldsymbol{,t+4}}$ | 0.004 | 0.004 | 0.004 | 0.000 |
|  | 0.010 | 0.010 | 0.010 | 0.010 |
| **Intercept** | 0.193*** | 0.191*** | 0.190*** | 0.198*** |
|  | 0.036 | 0.036 | 0.036 | 0.036 |
| **Adjusted R^2^** | 0.040 | 0.042 | 0.038 | 0.037 |
| **Observations** | 760 | 760 | 760 | 760 |

This table reports the result of the dependent variable ${BOND}_{t,t+4}$.

Robust *t*-statistics are displayed in parentheses. *** indicates the 1% significance level; ** indicates the 5% significance level; * indicates the 10% significance level.
