# Supplementary material for: Cross-asset momentum and the hybrid fund transmission mechanism in China’s stock and bond markets
Source: PLoS One. 2024 Mar 21;19(3):e0300781. doi: 10.1371/journal.pone.0300781 (PMC10956777; doi:10.1371/journal.pone.0300781)
Supplement: Attachment — Submitted filename: report.docx [file pone.0300781.s003.docx]

Review Report

Paper: **Cross-asset momentum and the hybrid fund transmission mechanism in
China’s stock and bond markets**

Comments:

The idea of the paper is interesting, the paper is quite well-written. However, there are several comments:

- The authors used many arguments without any reference, for example: “July 2022,
the total market capitalization of China’s A-share market reached approximately 12
trillion US dollars, making it one of the largest stock markets in the world. Additionally,
the bond market size in China has grown substantially, reaching up to 20 trillion US
dollars, becoming the second largest bond market globally”, and many other arguments in the Introduction parts.

- The data sources for all figures need to be cited.

- I cannot see any content of your Panels A, B in the introduction part. Are they the Figures 1A, 1B at the end of your paper?

- The authors should structure the Introduction part: There is no findings, implication here.
