# Supplementary material for: Cross-asset momentum and the hybrid fund transmission mechanism in China’s stock and bond markets
Source: PLoS One. 2024 Mar 21;19(3):e0300781. doi: 10.1371/journal.pone.0300781 (PMC10956777; doi:10.1371/journal.pone.0300781)
Supplement: Attachment — Submitted filename: Response to Reviewers.docx [file pone.0300781.s004.docx]

**Reviewer #1:**

**The authors revised the paper based on comments, but I don't think that equations (11) to (13) can fully explain the intermediary role of hybrid funds.**

**For instance, in one hybrid fund, 60% of its total assets are invested in stocks. If these stocks yield a return of 50%, then the hybrid fund will generate a return of 30%. Since stock prices impact returns for hybrid funds due to their inclusion as components within them, it seems rather intuitive.**

**Therefore, testing the intermediary effect of hybrid fund returns is meaningless; instead, examining shareholding ratios may be more persuasive.**

We appreciate the valuable comments from the Reviewer.

**Firstly,** We appreciate the Reviewer's suggestion to incorporate shareholding data, which is accurate and intuitive. As recommended, we acknowledge the work of Bradrania et al. (2023) (*Bradrania, R., & Wu, W. (2023). Foreign institutions, local investors and momentum trading. Journal of Empirical Finance*.), who successfully utilized unique daily frequency shareholding data from the Australian stock market. Their findings underscored the greater significance of institutional investors' momentum trading behavior compared to individual investors. However, their study faced constraints in micro-level trading data availability, limiting the daily frequency of shareholding data from 2007 to 2014.

In the current landscape, most existing literature relies on quarterly frequency data for shareholding, where funds disclose the proportion of stocks and bonds in their portfolios through quarterly financial reports. While we have considered incorporating shareholding data as explanatory variables to explore the transmission mechanism of cross-asset momentum, this approach presents challenges associated with insufficient data sample size and availability issues.

In terms of data sample size, our study relies on weekly frequency data, deliberately avoiding the utilization of lower-frequency monthly or quarterly data. This decision is underpinned by the fact that developed financial markets, such as the United States, offer a historically longer market data window. Even with lower-frequency data, we ensure a sufficient sample size for statistical power. In contrast, China, as an emerging market, has a shorter historical market data window. Using quarterly frequency data would result in a dearth of sample points and alignment issues with indicators.

To illustrate, when examining the performance of a specific type of fund index from 2015 to 2023 (a period marked by explosive growth in hybrid funds in China), the corresponding fund shareholding data provides a mere 32 sample points in the time dimension, calculated as eight years multiplied by four quarters. In contrast, the weekly frequency asset return series contains over a hundred sample points during the same period.

Considering data availability, given our current accessible data, employing various fund indices as explanatory variables at the weekly frequency represents a balanced choice. (Macro indicators, used as control variables, are only available at quarterly or monthly frequencies, and we managed these data using interpolation.) Looking ahead, delving into the cross-asset momentum trading behavior of China's institutional investors could be an intriguing avenue for future research, particularly by obtaining monthly or even higher-frequency shareholding data.

**Furthermore**, we delved into other pertinent fund-related indicators, drawing inspiration from Pitkäjärvi et al. (2020) (*Pitkäjärvi, A., Suominen, M., & Vaittinen, L. (2020). Cross-asset signals and time series momentum. Journal of Financial Economics.*), where we considered fund flow. However, this indicator presented two challenges: Firstly, the impact of fund flows on the stock and bond market is too intuitive. Secondly, when utilizing fund flows or the change rate of fund flows as variables, we observed a lack of statistical significance, pointing to the insufficient explanatory power of these indicators. Moreover, existing literature, including Pitkäjärvi et al. (2020), has extensively analyzed the relationship between fund flows and momentum. Replicating their research paradigm would lack innovation.

The hybrid fund indices employed in our study are comprehensive weighted indices settled by exchanges daily. Despite their authority and representativeness, an endogeneity issue arises, particularly concerning the "inclusion of stock market indices as components in fund indices." Nonetheless, our paper's results are distinctive, exploratory, and innovative. Furthermore, in equations (11) to (13), the introduction of instrumental variables partially mitigates concerns related to endogeneity.

**Finally,** In response to the reviewer's concern regarding the mediation analysis framework, we have introduced a moderation effect model to explore the moderating role of hybrid fund indices in the cross-asset momentum transmission process. This serves as an additional analysis to complement our study.

We formulated the following model to assess the moderating effect of fund indices in the transmission of bond market momentum to the stock market:

$${{STOCK}_{t,t+4}=\gamma_{0}+\gamma_{1}FUND}_{t-4,t-1}+\gamma_{2}{CUMBOND}_{t-52,t-1}+\gamma_{3}{FUND}_{t-4,t-1}\times{CUMBOND}_{t-52,t-1}$$

$$+\gamma_{4}{CUMSTOCK}_{t-52,t-1}+\gamma_{5}{FUND}_{t-4,t-1}\times{CUMSTOCK}_{t-52,t-1}$$

$+\gamma_{6}{SHIBOR}_{t,t+4}+{\gamma_{7}M2}_{t,t+4}+\gamma_{8}{CPI}_{t,t+4}+\gamma_{9}{PMI}_{t,t+4}+\varepsilon_{t}$	(A1)

In Equation A1, the dependent variable ${STOCK}_{t,t+4}$ represents the future one-month return of the stock market index. The ${FUND}_{t-4,t-1}$ represents the past one-month return of the fund index and the ${CUMBOND}_{t-52,t-1}$ signifies the cumulative return of the bond market over the past 52 weeks, reflecting momentum in bond market returns.

The term ${FUND}_{t-4,t-1}\times{CUMBOND}_{t-52,t-1}$ represents the interaction term between the fund index and the cumulative return of the bond market. This term indicates the moderating effect of hybrid fund indices on bond market momentum in the transmission to the stock market.

The control variable ${CUMSTOCK}_{t-52,t-1}$ is included for stock market momentum, while ${FUND}_{t-4,t-1}\times{CUMSTOCK}_{t-52,t-1}$ serves as the control variable for the fund index and stock market momentum interaction. Additional control variables encompass macro factors that could potentially influence the regression results.

The interpretation of Equation A1 unfolds as follows: Given the significant $\gamma_{2}$ for ${CUMBOND}_{t-52,t-1}$ (indicating a significant impact of bond market momentum on the stock market), our primary focus shifts to $\gamma_{3}$. A statistically significant $\gamma_{3}$ would signify that the fund indices play a moderating role in transmitting bond market momentum to the stock market.

To delve into specifics, when $\gamma_{2}$ is significantly positive, a concurrently significant positive $\gamma_{3}$ suggests that the fund indices amplify the positive impact of bond market momentum on the stock market. Conversely, a significantly negative $\gamma_{3}$ would indicate that hybrid fund indices impede the positive influence of bond market momentum on the stock market.

Concerns regarding the potential correlation between the moderating variable ${FUND}_{t-4,t-1}$ and the primary variable ${CUMBOND}_{t-52,t-1}$ may arise. The coefficient $\gamma_{3}$ of the interaction term ${FUND}_{t-4,t-1}\times{CUMSTOCK}_{t-52,t-1}$ could potentially reflect the indirect impact of the primary variable ${CUMBOND}_{t-52,t-1}$ on the dependent variable ${STOCK}_{t,t+4}$ (i.e., the influence of the square term of ${CUMBOND}_{t-52,t-1}$) rather than the moderation effect of ${FUND}_{t-4,t-1}$ itself.

We incorporate ${FUND}_{t-4,t-1}$ in Equation A1 to address this concern. The coefficient $\gamma_{1}$ of ${FUND}_{t-4,t-1}$ assists in interpreting the meaning of $\gamma_{3}$. If $\gamma_{1}$ is found to be insignificant, it implies that the primary variable ${CUMBOND}_{t-52,t-1}$ cannot indirectly influence the dependent variable ${STOCK}_{t,t+4}$ through its impact on ${FUND}_{t-4,t-1}$. In this scenario, we conclude that $\gamma_{3}$ reflects the moderation effect of hybrid fund indices on bond market momentum.

Conversely, if $\gamma_{1}$ is significant, we cannot dismiss the possibility that ${CUMBOND}_{t-52,t-1}$ indirectly affects ${STOCK}_{t,t+4}$ through its influence on ${FUND}_{t-4,t-1}$, and we cannot confirm the moderation effect of hybrid funds.

Table A1 presents the outcomes of Equation A1.

**Table A1. Result of the hybrid fund moderation on cross-asset momentum: from the bond to the stock market.**

| **Dependent variable:** $\boldsymbol{STOCK}_{\boldsymbol{t}\boldsymbol{,t+4}}$ | | | | |
| --- | --- | --- | --- | --- |
|  | **(1)** | **(2)** | **(3)** | **(4)** |
| $\boldsymbol{FUND}_{\boldsymbol{t}\boldsymbol{-4,t-1}}$ | **CSI Fund**  **Fund Index** | **Wind Partial**  **Stock**  **Fund Index** | **Wind Flexible**  **Fund Index** | **Wind Partial Bond**  **Fund Index** |
|  | 0.005 | 0.004 | -0.009 | 0.050 |
|  | 0.031 | 0.023 | 0.034 | 0.101 |
| $\boldsymbol{CUMBOND}_{\boldsymbol{t}\boldsymbol{-52,t-1}}$ | 0.154*** | 0.154*** | 0.157*** | 0.143*** |
|  | 0.039 | 0.046 | 0.047 | 0.046 |
| $\boldsymbol{FUND}_{\boldsymbol{t}\boldsymbol{-4,t-1}}\boldsymbol{\times}$  $\boldsymbol{CUMBOND}_{\boldsymbol{t}\boldsymbol{-52,t-1}}$ | 0.027** | 0.031** | 0.032** | 0.054* |
|  | 0.012 | 0.014 | 0.015 | 0.030 |
| $\boldsymbol{CUMSTOCK}_{\boldsymbol{t}\boldsymbol{-52,t-1}}$ | 0.002 | 0.002 | 0.002 | 0.002 |
|  | 0.004 | 0.004 | 0.004 | 0.004 |
| $\boldsymbol{FUND}_{\boldsymbol{t}\boldsymbol{-4,t-1}}\boldsymbol{\times}$  $\boldsymbol{CUMSTOCK}_{\boldsymbol{t}\boldsymbol{-52,t-1}}$ | 0.000 | 0.000 | 0.002 | -0.001 |
|  | 0.000 | 0.000 | 0.005 | 0.002 |
| $\boldsymbol{SHIBOR}_{\boldsymbol{t}\boldsymbol{,t+4}}$ | 0.001 | 0.002 | -0.002 | 0.011 |
|  | 0.113 | 0.112 | 0.112 | 0.113 |
| ${\boldsymbol{M}\boldsymbol{2}}_{\boldsymbol{t}\boldsymbol{,t+4}}$ | -0.587 | -0.593 | -0.535 | -0.703 |
|  | 0.547 | 0.547 | 0.547 | 0.547 |
| $\boldsymbol{CPI}_{\boldsymbol{t}\boldsymbol{,t+4}}$ | 0.213 | 0.148 | 0.160 | 0.147 |
|  | 0.284 | 0.284 | 0.284 | 0.285 |
| $\boldsymbol{PMI}_{\boldsymbol{t}\boldsymbol{,t+4}}$ | -0.001 | 0.010 | -0.008 | -0.014 |
|  | 0.140 | 0.139 | 0.140 | 0.139 |
| **Intercept** | -0.450 | -0.448 | -0.451 | -0.444 |
|  | 0.481 | 0.481 | 0.481 | 0.480 |
| **Adjusted R^2^** | 0.014 | 0.011 | 0.011 | 0.013 |
| **Observations** | 760 | 760 | 760 | 760 |

This table reports the result of the dependent variable ${STOCK}_{t,t+4}$.

Robust *t*-statistics are displayed in parentheses. *** indicates the 1% significance level; ** indicates the 5% significance level; * indicates the 10% significance level.

Firstly, for all four types of hybrid funds, the insignificance of $\gamma_{1}$ suggests that the past one-month return of the fund index does not forecast the future one-month return of the stock market index. This dismisses the possibility of the primary variable ${CUMBOND}_{t-52,t-1}$ indirectly influencing the dependent variable ${STOCK}_{t,t+4}$ by impacting ${FUND}_{t-4,t-1}$ (as indicated in the main results Table 9, where ${FUND}_{t,t+4}$ and ${STOCK}_{t,t+4}$ show significant correlation as they pertain to the same period). Furthermore, the coefficients for the control variables ${CUMSTOCK}_{t-52,t-1}$ and ${FUND}_{t-4,t-1}\times{CUMSTOCK}_{t-52,t-1}$ are also deemed insignificant.

Secondly, the significantly positive $\gamma_{2}$ indicates a favorable impact of bond market momentum on the stock market.

Lastly, the significantly positive $\gamma_{3}$ across all four types of hybrid funds signifies that in the process of bond market momentum transmission to the stock market, these hybrid fund indices amplify the positive impact of bond market momentum on the stock market.

Notably, the Wind Partial Bond Fund Index exhibits the most substantial moderating effect on bond market momentum, with a coefficient of 0.054. Intuitively, funds of this type maintain the highest bond allocation. Funds with higher bond allocations experience more excellent benefits during consistent bond price increases. Consequently, these funds rebalance their stock-bond asset ratios, allocating more capital to the stock market.

Similarly, we formulated the following model to assess the moderating effect of fund indices in the transmission of stock market momentum to the bond market:

$${{BOND}_{t,t+4}=\gamma_{0}+\gamma_{1}FUND}_{t-4,t-1}+\gamma_{2}{CUMSTOCK}_{t-52,t-1}+\gamma_{3}{FUND}_{t-4,t-1}\times{CUMSTOCK}_{t-52,t-1}$$

$$+\gamma_{4}{CUMBOND}_{t-52,t-1}+\gamma_{5}{FUND}_{t-4,t-1}\times{CUMBOND}_{t-52,t-1}$$

$+\gamma_{6}{SHIBOR}_{t,t+4}+{\gamma_{7}M2}_{t,t+4}+\gamma_{8}{CPI}_{t,t+4}+\gamma_{9}{PMI}_{t,t+4}+\varepsilon_{t}$	(A2)

In Equation A2, the dependent variable ${BOND}_{t,t+4}$ represents the future one-month return of the bond market index. The ${FUND}_{t-4,t-1}$ signifies the past one-month return of the fund index and the ${CUMSTOCK}_{t-52,t-1}$ reflects the cumulative return of the stock market over the past 52 weeks, indicative of momentum in stock market returns.

The term ${FUND}_{t-4,t-1}\times{CUMSTOCK}_{t-52,t-1}$ represents the interaction term between the past one-month return of the fund index and the cumulative return of the stock market over the past 52 weeks. This term signifies the moderating effect of the hybrid fund index on stock market momentum in the transmission to the bond market.

The variable ${CUMBOND}_{t-52,t-1}$ serves as the control variable for bond market momentum and ${FUND}_{t-4,t-1}\times{CUMBOND}_{t-52,t-1}$ functions as the control variable for the fund index and bond market momentum interaction. The remaining variables serve as control variables for macro factors.

The interpretation of Equation A2 is as follows: Given the significant $\gamma_{2}$ for ${CUMSTOCK}_{t-52,t-1}$ (indicating a significant impact of stock market momentum on the bond market), the significance of the coefficient $\gamma_{3}$ in the interaction term ${FUND}_{t-4,t-1}\times{CUMSTOCK}_{t-52,t-1}$ implies that the fund indices play a moderating role in transmitting stock market momentum to the bond market.

To be more specific, when $\gamma_{2}$ is significantly negative, significant and positive $\gamma_{3}$ suggests that the fund index mitigates the negative impact of stock market momentum on the bond market. Conversely, if $\gamma_{3}$ is significantly negative, it indicates that hybrid fund indices amplify the negative influence of stock market momentum on the bond market.

Table A2 reports the results of Equation A2.

**Table A2 Result of the hybrid fund moderation on cross-asset momentum: from the stock to the bond market.**

| **Dependent variable:** $\boldsymbol{BOND}_{\boldsymbol{t}\boldsymbol{,t+4}}$ | | | | |
| --- | --- | --- | --- | --- |
|  | **(1)** | **(2)** | **(3)** | **(4)** |
| $\boldsymbol{FUND}_{\boldsymbol{t}\boldsymbol{-4,t-1}}$ | **CSI Fund**  **Fund Index** | **Wind Partial**  **Stock**  **Fund Index** | **Wind Flexible**  **Fund Index** | **Wind Partial Bond**  **Fund Index** |
|  | -0.001 | -0.001 | -0.002 | -0.004 |
|  | 0.002 | 0.002 | 0.002 | 0.007 |
| $\boldsymbol{CUMSTOCK}_{\boldsymbol{t}\boldsymbol{-52,t-1}}$ | -0.001*** | -0.003*** | -0.003*** | -0.002*** |
|  | 0.000 | 0.001 | 0.001 | 0.000 |
| $\boldsymbol{FUND}_{\boldsymbol{t}\boldsymbol{-4,t-1}}\boldsymbol{\times}$  $\boldsymbol{CUMSTOCK}_{\boldsymbol{t}\boldsymbol{-52,t-1}}$ | -0.009** | -0.008*** | -0.008** | -0.002** |
|  | 0.003 | 0.002 | 0.003 | 0.001 |
| $\boldsymbol{CUMBOND}_{\boldsymbol{t}\boldsymbol{-52,t-1}}$ | -0.005* | -0.004* | -0.005* | 0.006* |
|  | 0.005 | 0.003 | 0.004 | 0.003 |
| $\boldsymbol{FUND}_{\boldsymbol{t}\boldsymbol{-4,t-1}}\boldsymbol{\times}$  $\boldsymbol{CUMBOND}_{\boldsymbol{t}\boldsymbol{-52,t-1}}$ | -0.001 | -0.002 | -0.001 | -0.001 |
|  | 0.001 | 0.002 | 0.001 | 0.001 |
| $\boldsymbol{SHIBOR}_{\boldsymbol{t}\boldsymbol{,t+4}}$ | -0.025*** | -0.024*** | -0.028*** | -0.030*** |
|  | 0.007 | 0.007 | 0.008 | 0.008 |
| ${\boldsymbol{M}\boldsymbol{2}}_{\boldsymbol{t}\boldsymbol{,t+4}}$ | -0.006 | -0.005 | -0.005 | -0.003 |
|  | 0.039 | 0.039 | 0.040 | 0.040 |
| $\boldsymbol{CPI}_{\boldsymbol{t}\boldsymbol{,t+4}}$ | 0.022 | 0.022 | 0.023 | 0.019 |
|  | 0.021 | 0.021 | 0.021 | 0.021 |
| $\boldsymbol{PMI}_{\boldsymbol{t}\boldsymbol{,t+4}}$ | 0.004 | 0.004 | 0.004 | 0.000 |
|  | 0.010 | 0.010 | 0.010 | 0.010 |
| **Intercept** | 0.193*** | 0.191*** | 0.190*** | 0.198*** |
|  | 0.036 | 0.036 | 0.036 | 0.036 |
| **Adjusted R^2^** | 0.040 | 0.042 | 0.038 | 0.037 |
| **Observations** | 760 | 760 | 760 | 760 |

This table reports the result of the dependent variable ${BOND}_{t,t+4}$.

Robust *t*-statistics are displayed in parentheses. *** indicates the 1% significance level; ** indicates the 5% significance level; * indicates the 10% significance level.

Firstly, across all four types of hybrid funds, the insignificance of $\gamma_{1}$ suggests that the past one-month return of the fund index does not forecast the future one-month return of the bond market index. This dismisses the possibility of the primary variable ${CUMSTOCK}_{t-52,t-1}$ indirectly influencing the dependent variable ${BOND}_{t,t+4}$ by impacting ${FUND}_{t-4,t-1}$. The coefficient for the control variable ${CUMBOND}_{t-52,t-1}$ is positive at the 10% significance level, but the coefficient for the interaction term ${FUND}_{t-4,t-1}\times{CUMBOND}_{t-52,t-1}$ is deemed insignificant.

Secondly, the significantly negative $\gamma_{2}$ suggests a negative impact of stock market momentum on the bond market.

Lastly, $\gamma_{3}$ is significantly negative for all four types of hybrid funds, indicating that in the process of stock market momentum transmission to the bond market, hybrid fund indices amplify the negative impact of stock market momentum on the bond market.

In contrast to the findings reported in Table A1, it is evident that the Wind Partial Bond Fund Index demonstrates the least impactful moderating effect on stock market momentum at this juncture. This is indicated by the smallest absolute coefficient value, primarily attributed to these funds maintaining the lowest allocation to stocks. In periods of consistent stock price increases, these funds encounter restricted gains. Furthermore, due to the regulatory constraints associated with portfolio allocation ratios for these funds, their ability to enhance stock holdings by decreasing bond holdings is limited.

The above comprises our response to the concerns raised by the Reviewer. We have incorporated the additional empirical results into the appendix of the revised manuscript, distinctly highlighted in red.

Once again, we express our gratitude for the Reviewer's valuable insights. We trust that our responses effectively address the Reviewer's inquiries and contribute to the continual improvement of the paper.
